# Impact of socioeconomic status on tumor stage, molecular subtype distribution, treatment adherence, and survival outcomes in breast cancer

**DOI:** 10.3389/fonc.2026.1824744

**Published:** 2026-05-08

**Authors:** Ting Li, Huan Liu, Shuang Ma, Li Guo, Jinping Li

**Affiliations:** 1 Clinical Medical School, Ningxia Medical University, Yinchuan, Ningxia, China; 2 Department of Surgical Oncology, General Hospital of Ningxia Medical University, Yinchuan, Ningxia, China

**Keywords:** breast cancer, health disparities, molecular subtype, socioeconomic status, stage of tumor, survival rates, treatment compliance

## Abstract

**Background:**

Socioeconomic status (SES) is a key determinant of cancer outcomes, influencing access to screening, early diagnosis, treatment adherence, and survival. Socioeconomic disparities in breast cancer may contribute to differences in tumor biology, stage at diagnosis, and prognosis.

**Purpose:**

This study aimed to evaluate the impact of socioeconomic status on tumor stage at diagnosis, molecular subtype distribution, treatment utilization, adherence, and survival outcomes among patients with breast cancer.

**Methodology:**

A retrospective cohort study was conducted using hospital cancer registries and electronic medical records from a tertiary oncology center. A total of 800 patients diagnosed with primary invasive breast cancer were included and categorized into low (n = 300), middle (n = 300), and high (n = 200) SES groups based on income, education, and insurance status. Clinical and pathological variables were collected, including tumor stage, molecular subtype, treatment modalities, adherence patterns, and diagnostic delays. Survival outcomes were assessed using overall survival (OS) and disease-free survival (DFS) with a minimum follow-up of 72 months. Chi-square and ANOVA tests examined group differences, while multivariate logistic regression and Cox proportional hazards models identified predictors of late-stage diagnosis and survival outcomes.

**Results:**

Patients with low SES were significantly more likely to present with late-stage disease (49.3%) than those with high SES (25.0%) (p < 0.001). Low SES patients also showed more aggressive tumor characteristics, including larger tumor size, lymph node involvement, higher tumor grade, and elevated Ki-67 levels (p < 0.01). Triple-negative breast cancer was more prevalent in the low SES group (29.3%). Treatment adherence and completion rates were lower among low SES patients, accompanied by greater diagnostic and treatment delays. Survival analysis showed poorer outcomes in low SES patients, with reduced median OS (64 vs. 88 months) and DFS (50 vs. 80 months). Multivariate analysis confirmed low SES as an independent predictor of late-stage diagnosis (OR 2.45) and poorer survival (HR 1.72 for OS; HR 1.60 for DFS).

**Conclusion:**

Socioeconomic disparities significantly influence breast cancer stage at diagnosis, tumor characteristics, treatment adherence, and survival. Addressing socioeconomic barriers and improving equitable access to cancer care are essential to reduce disparities in breast cancer outcomes.

## 1 Introduction

Breast cancer is the most prevalent diagnosed malignancy in women and a major cause of cancer-related death in the global sphere. It imposes a substantial global health burden, affecting both population health and healthcare systems. The latest world cancer statistics indicate that breast cancer is the most prevalent cancer diagnosed globally, and among 2.6 million new cases and 670,000 fatalities in 2022, breast cancer continues to pose a substantial burden and show a rising pattern of disparities in diagnosis, access to care, and survival among populations worldwide (1). Irrespective of significant progress in the field of early cancer detection and specific treatment methods, significant differences in breast cancer diagnosis, treatment, and survival continue to exist among socioeconomic groups.

Socioeconomic status (SES), which is often determined by income, education, occupation, and neighborhood conditions, is an important factor in access to healthcare services and cancer outcomes. The disadvantaged SES is often linked with lower attendance at breast cancer screening programs and late diagnosis. It has been revealed that poor populations tend to have more frequent advanced tumors, and it is one of the main reasons that lead to worse survival rates (2). Population-based research has demonstrated that the disparity in breast cancer survival between regions and populations is impressive due to the socioeconomic inequalities (3). Evidence from population-based multi-ethnic studies further indicates that both neighborhood- and individual-level socioeconomic factors significantly influence adherence to breast cancer screening practices, with economically disadvantaged groups demonstrating lower compliance rates. Reduced screening participation ultimately increases the likelihood of diagnosis at advanced stages, which remains one of the strongest predictors of breast cancer mortality (4).

Beyond the diagnostic stage, SES can also affect tumor biology and molecular subtype distribution. Breast cancer is a heterogeneous disorder, which has molecular subtypes like hormone receptor-positive, HER2-positive, and triple-negative tumors with various prognoses and treatments. It has been shown that socioeconomic factors can affect the occurrence of certain aggressive tumor subtypes, with possible correlations among social determinants, environmental exposures, and tumor characteristics (5). Moreover, it has been reported that there are discrepancies in mortality across molecular subtypes compared with the general population, suggesting an interplay between biological and socioeconomic factors (6).

Socioeconomic inequalities also influence treatment accessibility and compliance. Patients who are of a lower socioeconomic status are usually subjected to some obstacles in the form of financial constraints, health access, and health literacy, which may result in delayed treatment or incomplete treatment. Research revealed that socioeconomic aspects had a great impact on treatment patterns and survival in metastatic breast cancer (7). In aggressive breast cancer, like triple-negative, any inequality in access to treatment can further deteriorate the patient outcomes (8).

Moreover, healthcare access disparities and neighborhood-level deprivation are the factors that lead to differences in the mortality of breast cancer. As it turns out, women inhabiting socioeconomically disadvantaged societies were found to be at a greater risk of mortality than those in more prosperous ones (9). The disparity in access to more sophisticated diagnostic technologies and genomic testing, including Oncotype DX, could also play a role in the survival differences between breast cancer patients (10). Such differences might be especially noticeable in younger patients, in whom socioeconomic barriers have been associated with later diagnosis and worse survival (11).

Even though a lot of research has been done on socioeconomic variations in breast cancer outcomes, a majority of them have concentrated on single variables, including stage of diagnosis or survival, respectively. There is a dearth of studies that have concurrently measured the associations among socioeconomic status, molecular subtype, treatment adherence, and survival within a single cohort. Thus, the proposed retrospective study is the research that will be used to explore how socioeconomic status affects the stage at diagnosis, molecular distribution of subtypes, treatment compliance, and patient survival in cases of breast cancer. Knowledge of such associations can be of great importance for understanding the mechanisms underlying socioeconomic disparities and can inform strategies to enhance equity in breast cancer care. We therefore hypothesized that lower socioeconomic status is an independent factor that leads to a more advanced stage at diagnosis, a greater number of aggressive molecular subtypes, worse treatment adherence and lower survival outcomes in breast cancer patients, which controls for clinical and demographic confounding factors.

## 2 Methodology

### 2.1 Study design and setting

The researchers performed a retrospective cohort study to investigate how socioeconomic status (SES) affects breast cancer patients. The researchers obtained clinical and pathological information through the institutional cancer registry and the electronic medical record system at a tertiary care oncology center. The research received approval to study all patients diagnosed between January 2020 and December 2025.

### 2.2 Study population

The study group included 800 female patients with histologically confirmed primary invasive breast cancer. The research excluded patients who had incomplete medical records or who had a history of previous malignancies or had developed metastatic disease from cancers that originated outside of breast tissue. Participants in the study were divided into three socioeconomic groups: 300 from the low group, 300 from the middle group, and 200 from the high group. The researchers developed their SES classification system by creating an index that combined data on household income, educational achievement, and health insurance coverage.

### 2.3 Definition of exposure

The socioeconomic status (SES) was measured using a composite index based on three dimensions: household income, educational attainment, and insurance status. A score was given to each of the domains (0–2), where a high number signified more desirable socioeconomic factors.

- The income level was divided into tertiles of regional income.
- Education was categorized as ≤high school, secondary, or tertiary education.
- Insurance status was categorized as either uninsured, partially insured or fully insured.

The total SES score was between 0 and 6 and was categorized into three:

- Low SES (score 0–2)
- Middle SES (score 3–4)
- High SES (score 5–6)

The three SES indicators were equally weighted due to the absence of standardized weighting criteria and to maintain methodological simplicity, an approach commonly adopted in epidemiological studies assessing socioeconomic gradients (12, 13). Construct validity of the SES index was assessed by examining its associations with related socioeconomic indicators (e.g., insurance coverage, education level, and rural residence), which showed consistent gradients across SES categories. This justifies the validity of composite SES classification that has been employed in this study.

### 2.4 Data collection and variables

The study collected participant demographic data, including age at diagnosis, urban or rural residence, and the Charlson Comorbidity Index (CCI) score for comorbidity burden. The study used the American Joint Committee on Cancer (AJCC) staging system, tumor stage, tumor size, lymph node involvement, histological grade, and Ki-67 proliferation index as tumor-related variables. The researchers used immunohistochemical analysis to classify molecular subtypes into three categories, which were hormone receptor–positive/HER2-negative (HR+/HER2–) and HER2-positive and triple-negative breast cancer. The study recorded surgical procedures, chemotherapy, endocrine therapy, anti-HER2-targeted therapy, and radiotherapy as treatment modalities. The study measured treatment adherence through three indicators, which included recommended therapy completion and chemotherapy or endocrine therapy non-adherence, and treatment delays that lasted more than eight weeks. The researchers assessed diagnostic delay using three variables: symptom-to-diagnosis interval, diagnosis-to-treatment interval, and total delay.

### 2.5 Outcome measures

The primary outcomes of the study consisted of diagnosis-stage assessment, which measured progression from early stages I and II to advanced stages III and IV, and survival outcomes. The study measured survival outcomes using two metrics: overall survival (OS) and disease-free survival (DFS). The study collected survival data during follow-up, which lasted a median of 72 months.

### 2.6 Covariates and confounding control

The researchers identified age, residence type (which includes urban and rural areas), comorbidity burden (which requires CCI scores of 3 or higher), tumor stage, molecular subtype, and treatment adherence as factors that could distort research outcomes. The researchers used multivariable statistical models to examine how socioeconomic status affects medical outcomes. The researchers used logistic regression models to analyze factors that contributed to late-stage diagnosis, while Cox proportional hazards models evaluated the impact on overall survival and disease-free survival.

### 2.7 Statistical analysis

The researchers used descriptive statistics to summarize demographic, clinical, and treatment-related data across different socioeconomic status (SES) groups. Categorical variables were analyzed using Chi-square tests, while continuous variables were assessed using analysis of variance (ANOVA). The SES index was treated as an ordinal categorical variable in all regression models. Multivariable logistic regression was performed to estimate adjusted odds ratios (ORs) for late-stage presentation. Cox proportional hazards regression analysis was conducted to calculate adjusted hazard ratios (HRs) for overall and disease-free survival. A two-sided p-value < 0.05 was considered statistically significant, and analyses were performed using standard statistical software.

Multivariable models were developed based on clinical relevance and prior literature. The final models included variables with a p value less than 0.10 in univariate analysis and those deemed as important clinically (age, tumor stage, molecular subtype, treatment adherence, residence and comorbidity burden). Cox regression proportional hazard assumption was tested with Schoenfeld residual and log-minus-log survival plot and none of the significant violations were found. Standard diagnostic measures, such as variance inflation factors (VIFs), were used to assess model fit and multicollinearity.

### 2.8 Ethical considerations

The study obtained institutional ethical approval, while patient confidentiality will be protected through anonymized data processing, which complies with data protection laws. The study will bypass the need for informed consent because it functions as a retrospective analysis. This research was approved by the Ethics Committee of Ningxia Medical University, Approval Number: KYLL-2025-1620.

## 3 Results

### 3.1 Baseline cohort demographics and socioeconomic status distribution

**Figure 1:**
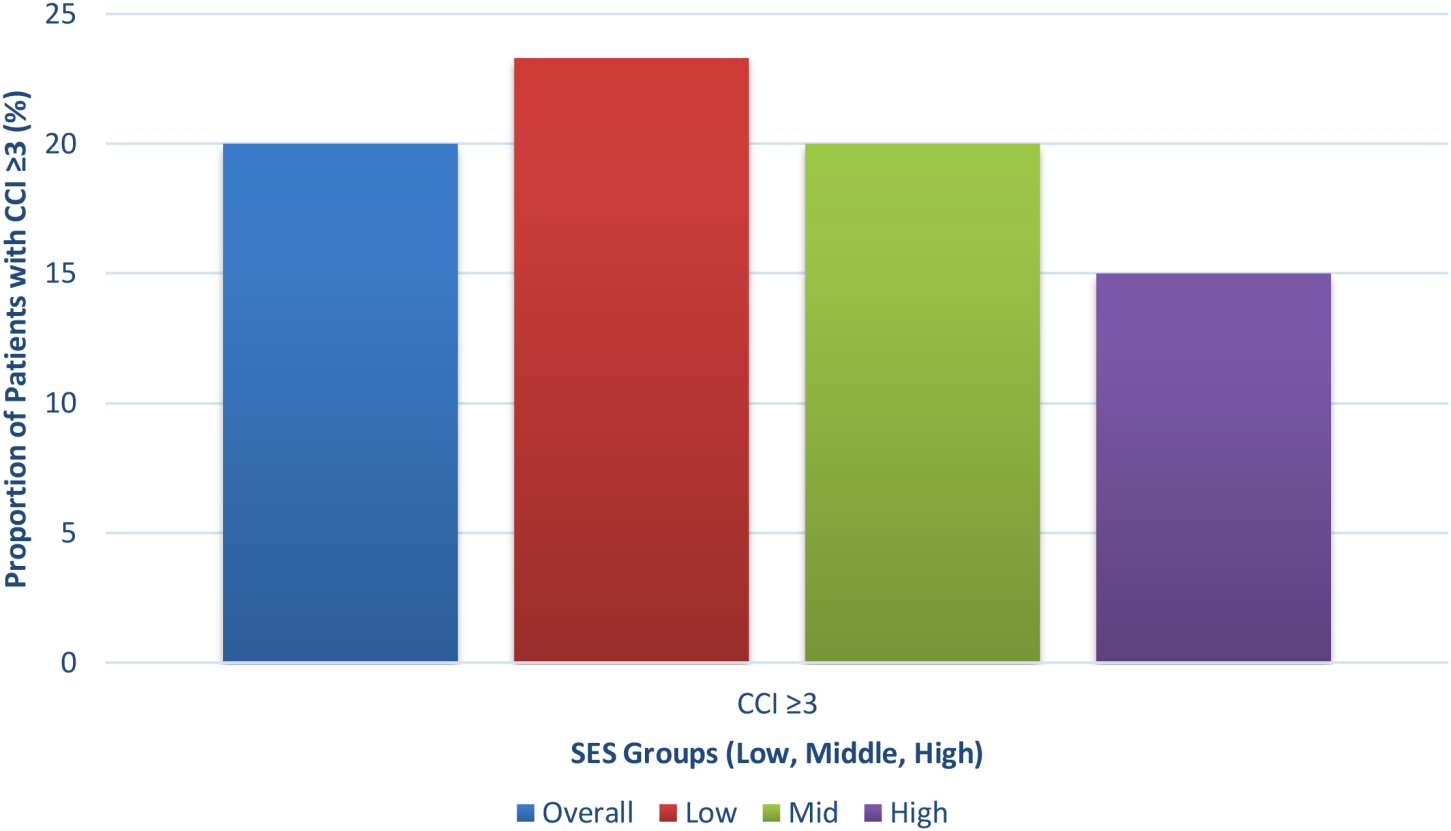
Distribution of comorbidity burden (Charlson Comorbidity Index ≥3) across socioeconomic status (SES) groups. The proportion of patients with higher comorbidity burden was greatest in the low SES group compared to middle and high SES groups.

The average age of the participants was 54.3 years, with an age range that extended from 43 to 66 years, and the study found a significant age difference between the three SES groups (p = 0.041) because high SES patients had an average age of 52.3 years, while low SES patients showed an average age of 55.1 years. All participants in the study were female who showed no considerable changes in their distribution across different SES groups (p = 0.88). People who lived in rural areas showed a strong association between their socioeconomic status and rural residency, with the highest occurrence in the low socioeconomic status group (60.0%) and the lowest in the high socioeconomic status group (20.0%) (p < 0.001). The study found that insurance coverage increased with higher SES, with 50.0% of low-SES individuals having insurance compared to 90.0% of high-SES individuals (p < 0.001). The low-SES group showed a high proportion of individuals who reached only high school, with 80.0% not completing their education beyond that level (p < 0.001). The study found that low SES patients had an increased occurrence of higher comorbidity burden (CCI ≥3) (p = 0.048) (**Table 1**, **Figure 1**).

**Table 1:** Cohort demographics & SES distribution.

Parameter	Total (n=800)	Low (n=300)	Mid (n=300)	High (n=200)	p-value
Age, mean ± SD	54.3 ± 11.8	55.1 ± 12.2	54.0 ± 11.5	52.3 ± 11.3	0.041
Female, n (%)	776 (97.0)	290 (96.7)	292 (97.3)	194 (97.0)	0.88
Rural Residence	340 (42.5)	180 (60.0)	120 (40.0)	40 (20.0)	<0.001
Insurance Coverage	540 (67.5)	150 (50.0)	210 (70.0)	180 (90.0)	<0.001
Education ≤High School	380 (47.5)	240 (80.0)	110 (36.7)	30 (15.0)	<0.001

### 3.2 Tumor stage at diagnosis stratified by socioeconomic status

**Figure 2:**
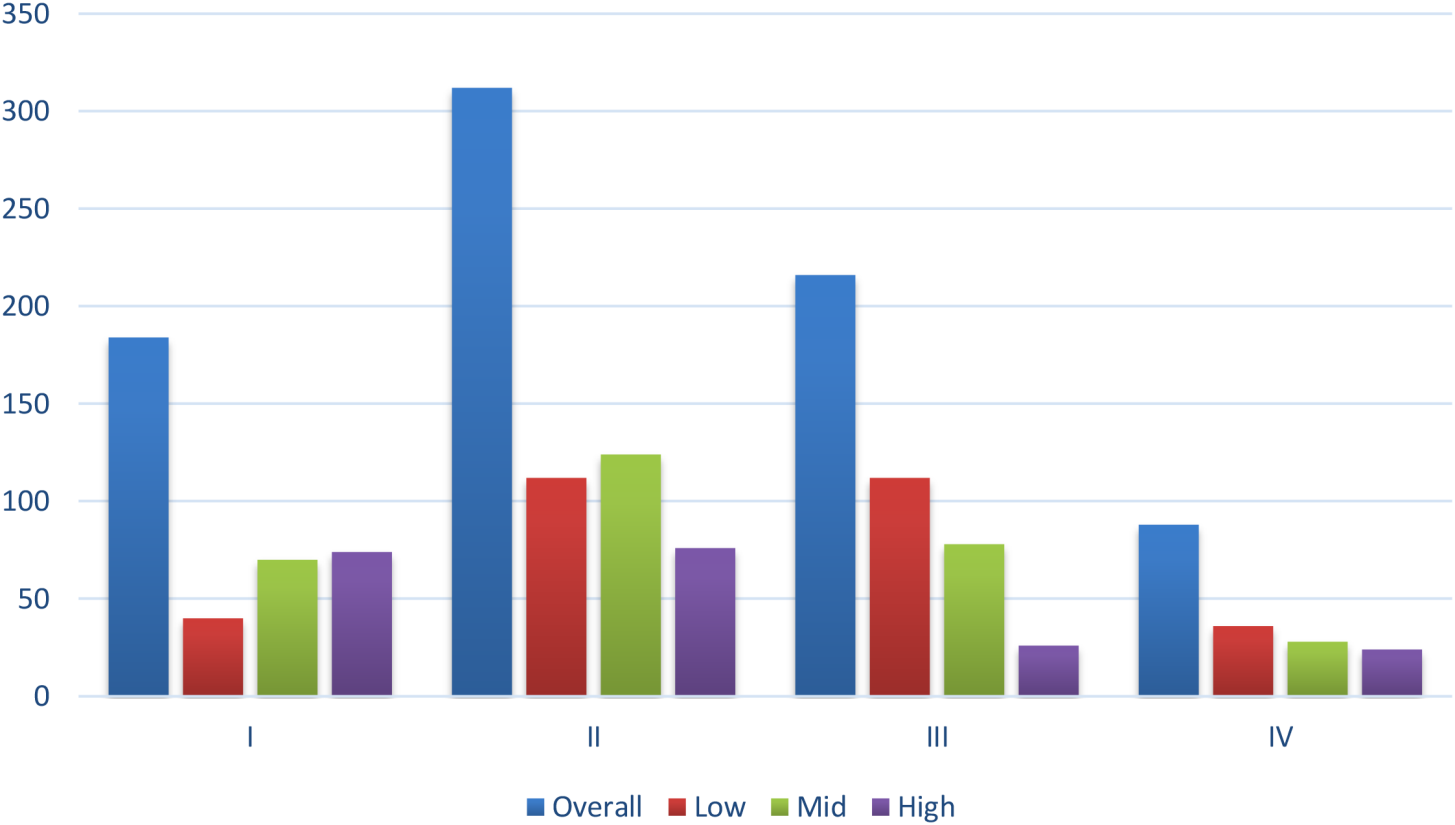
Stage at diagnosis by SES. Patients in the low SES group had a higher proportion of late-stage (III–IV) disease, while high SES patients were more frequently diagnosed at early stages (I–II).

Overall, 62.0% of patients were diagnosed at an early stage (I–II), while 38.0% presented with late-stage disease (III–IV). The stage at diagnosis showed a strong association with socioeconomic status, with the relationship statistically significant (p < 0.001). The high socioeconomic status group presented the highest Stage I disease rate at 37.0%, while they showed the lowest Stage III disease rate at 13.0%. The low SES group showed a substantial decrease in Stage I tumor cases 13.3% and an increase in advanced Stage III disease cases, 37.3%. Patients with high socioeconomic status had the highest early-stage diagnosis rate, at 75.0%, exceeding the middle-class rate of 64.7% and the low socioeconomic status rate of 50.7%. Low socioeconomic status patients showed almost double the late-stage presentation rate, which reached 49.3%, compared to the high socioeconomic status group, which had 25.0% of patients presenting at advanced disease stages (**Table 2**, **Figure 2**).

**Table 2:** Stage at diagnosis by SES.

Stage	Overall	Low	Mid	High	p-value
Early (I–II)	496 (62.0)	152 (50.7)	194 (64.7)	150 (75.0)	
Late (III–IV)	304 (38.0)	148 (49.3)	106 (35.3)	50 (25.0)	<0.001

### 3.3 Tumor pathological characteristics by socioeconomic status

There were more aggressive tumor characteristics in Low SES patients, such as a greater proportion of tumors 5 cm or bigger (29.3% vs 14.0% in high SES). Lymph node positivity was also highest in low SES (42.7%), compared to middle (40.0%) and high SES groups (28.0%; p = 0.002). High-grade tumors (Grade 3) were more common in low (34.7%) and middle SES (32.0%) than in high SES patients (16.0%; p < 0.001). Similarly, elevated Ki-67 (≥20%) was more frequent in low (52.0%) and middle (50.0%) SES groups than in high SES groups (33.0%; p < 0.001). In general, these results show that the lower socioeconomic groups have more aggressive tumor biology (**Table 3**).

**Table 3:** Tumor characteristics by SES.

Feature	Low	Mid	High	p-value
Tumor ≥5 cm	88 (29.3)	76 (25.3)	28 (14.0)	<0.001
Node Positive	128 (42.7)	120 (40.0)	56 (28.0)	0.002
Grade 3	104 (34.7)	96 (32.0)	32 (16.0)	<0.001
Ki-67 ≥20%	156 (52.0)	150 (50.0)	66 (33.0)	<0.001

### 3.4 Molecular subtype distribution across socioeconomic status groups

The distribution of molecular subtypes was significantly associated with socioeconomic status (p = 0.011). The most common in all groups was HR+/HER2 which was not as common among low SES patients. HER2-positive tumors did not vary significantly among groups. Conversely, the low SES group had a higher proportion of triple-negative breast cancer (29.3%), in comparison with middle (23.3) and high SES (21.0) groups, which points to a greater proportion of aggressive subtypes and worse outcomes in disadvantaged patients (**Table 4**).

**Table 4:** Molecular subtype distribution.

Subtype	Low	Mid	High	p-value
HR+/HER2–	138 (46.0)	168 (56.0)	114 (57.0)	–
HER2+	74 (24.7)	62 (20.7)	44 (22.0)	–
Triple Negative	88 (29.3)	70 (23.3)	42 (21.0)	0.011

### 3.5 Treatment modalities utilization by socioeconomic status

Low SES patients underwent surgical management at an 86.7% rate, which fell short of the 95.3% rate for middle SES patients and the 95.0% rate for high SES patients (p < 0.001). The low SES group showed the highest chemotherapy usage rate at 66.0%, which decreased with higher SES (p = 0.003) because they presented with more advanced disease. Endocrine therapy uptake among eligible patients showed a clear socioeconomic gradient, with low-SES patients (45.3%) having far lower uptake than middle-SES (62.0%) and high-SES (71.0%) patients (p < 0.001). HER2-positive cases showed high rates of anti-HER2 therapy, with no statistically significant differences across groups (p = 0.12). The use of radiation therapy increased with SES: low-SES patients showed 44.7% use, and high-SES patients showed 64.0% (p < 0.001), suggesting possible gaps in access to oncological treatment (**Table 5**).

**Table 5:** Treatment modalities.

Treatment	Low	Mid	High	p-value
Surgery	260 (86.7)	286 (95.3)	190 (95.0)	<0.001
Chemotherapy	198 (66.0)	180 (60.0)	102 (51.0)	0.003
Endocrine Therapy*	136 (45.3)	186 (62.0)	142 (71.0)	<0.001
Anti-HER2 (HER2+)	62/74 (83.8)	58/62 (93.5)	40/44 (90.9)	0.12
Radiation	134 (44.7)	158 (52.7)	128 (64.0)	<0.001

Endocrine therapy*=Hormone therapy.

### 3.6 Treatment adherence patterns by socioeconomic status

Patients with low socioeconomic status had the lowest treatment completion rate at 52%, while middle- and high-socioeconomic status groups achieved 68% and 75%, respectively (p < 0.001). The low socioeconomic status group had more than double the chemotherapy non-compliance rate (21.3%) compared with the high socioeconomic status group (9.0%) (p = 0.002). Low socioeconomic status patients showed higher endocrine therapy non-adherence rates, 29.3%, when compared to middle, 18.0%, and high SES groups, 14.0% (p < 0.001). The low socioeconomic status group showed treatment delays extending beyond 8 weeks at 36.7%, while the middle and high socioeconomic status groups showed delays at 26.0% and 14.0%, respectively (p < 0.001). The research results show that patients from lower socioeconomic backgrounds face major hurdles that prevent them from receiving essential cancer treatment (**Table 6**).

**Table 6:** Treatment adherence.

Metric	Low	Mid	High	p-value
Completed All Therapy	156 (52.0)	204 (68.0)	150 (75.0)	<0.001
Chemo Non-Adherence	64 (21.3)	42 (14.0)	18 (9.0)	0.002
Endocrine Non-Adherence	88 (29.3)	54 (18.0)	28 (14.0)	<0.001
Treatment Delay >8 wks	110 (36.7)	78 (26.0)	28 (14.0)	<0.001

### 3.7 Diagnostic and treatment delays across socioeconomic status

Low SES patients had the longest delays from symptom onset to diagnosis (11.2 ± 5.8 weeks) compared to middle (8.4 ± 4.9) and high SES groups (6.5 ± 3.5; p < 0.001). A similar trend was observed for diagnosis-to-treatment intervals (7.8 ± 3.9 vs 5.6 ± 3.1 vs 4.2 ± 2.8 weeks; p < 0.001). Overall, total delay was greatest in the low SES group (19.0 ± 8.2 weeks), nearly 8 weeks longer than in high SES patients (10.7 ± 6.1; p < 0.001). These findings indicate systemic barriers to timely care among disadvantaged populations, contributing to later-stage presentation and poorer outcomes (**Table 7**).

**Table 7:** Diagnostic & treatment delays (Mean ± SD).

Delay	Low	Mid	High	p-value
Symptom → Diagnosis	11.2 ± 5.8	8.4 ± 4.9	6.5 ± 3.5	<0.001
Diagnosis → Treatment	7.8 ± 3.9	5.6 ± 3.1	4.2 ± 2.8	<0.001
Total Delay	19.0 ± 8.2	14.0 ± 7.2	10.7 ± 6.1	<0.001

### 3.8 Survival outcomes by socioeconomic status - 72-month follow-up

**Figure 3:**
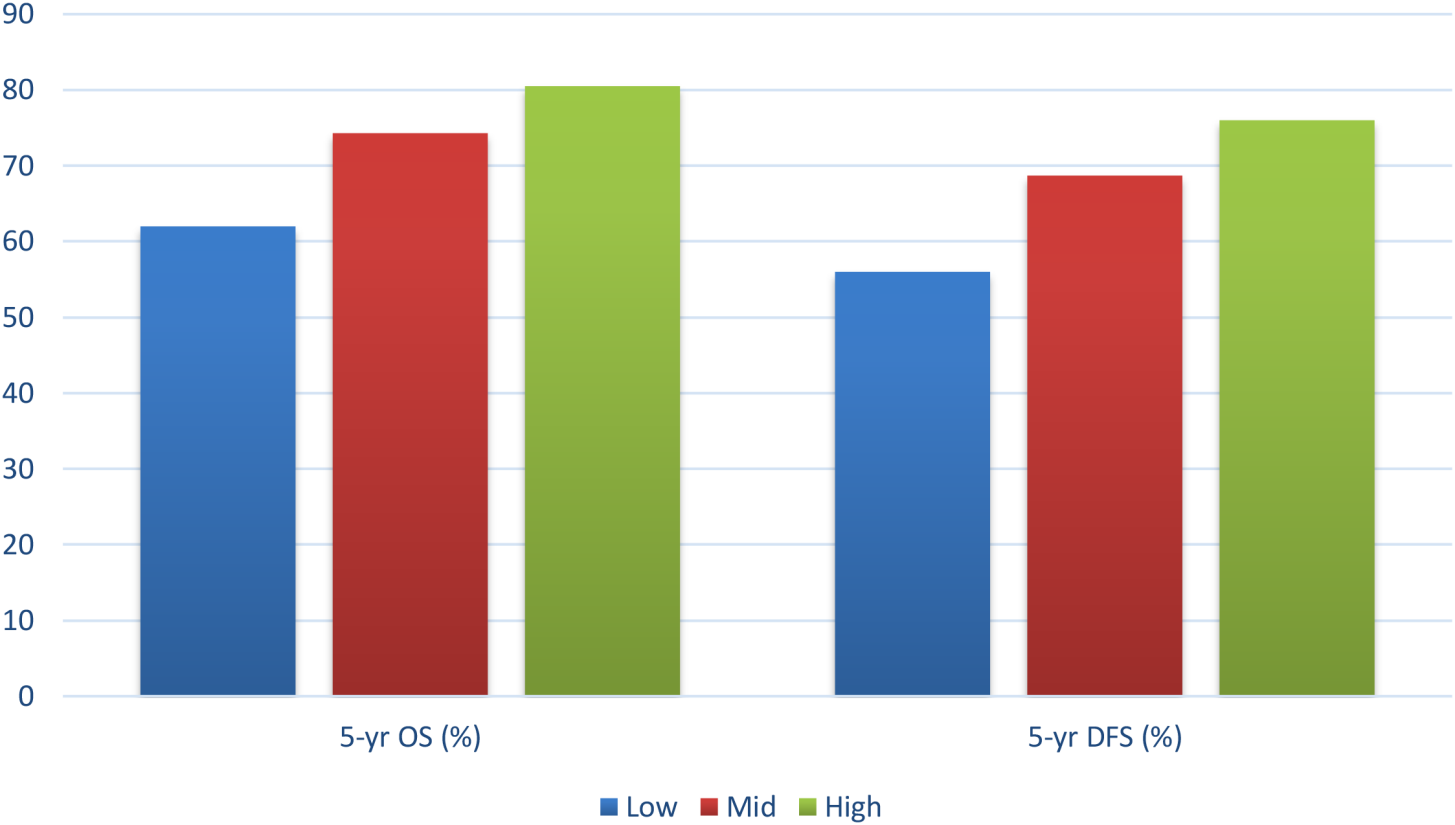
Survival outcomes. Kaplan–Meier survival curves demonstrating overall survival (OS) and disease-free survival (DFS) across SES groups over a 72-month follow-up period. Lower SES is associated with significantly reduced survival outcomes.

The survival (OS) in relation to socioeconomic status (low SES group 64 months to high SES group 88 months) was improved (log-rank p < 0.001). The same trend was observed in disease-free survival (DFS) whereby low SES patients had a median DFS of 50 months and high SES patients had a median DFS of 80 months (p < 0.001). OS rate at five years stood at 62.0, 74.3 and 80.5, respectively, in low, middle and high SES, and five-year DFS was 56.0, 76.0, respectively. These results show that socioeconomically disadvantaged patients have poorer long-term outcomes, probably because they were diagnosed later, their treatment is delayed, and adherence is lower (**Table 8**). Kaplan-Meier survival curves were constructed to compare overall survival (OS) and disease-free survival (DFS) across SES groups, demonstrating significantly poorer survival among patients with low socioeconomic status (**Figure 3**).

**Table 8:** Survival outcomes.

Outcome	Low	Mid	High	Log-rank p
Median OS (months)	64	78	88	<0.001
Median DFS (months)	50	68	80	<0.001

### 3.9 Multivariable logistic regression predicting late-stage diagnosis (III/IV)

Low socioeconomic status (SES) was strongly associated with increased odds of late-stage presentation (adjusted OR 2.45; 95% CI 1.70–3.52; p < 0.001) compared with high SES, while middle SES also demonstrated elevated risk (OR 1.45; 95% CI 1.02–2.05; p = 0.039). Residents of rural areas showed an independent tendency to develop late-stage disease (OR 1.60; 95% CI 1.15–2.24; p = 0.005), indicating that people from different geographic areas have varying levels of access to early diagnostic services. The diagnostic and treatment process required additional time, resulting in an 8% increase in the probability of late-stage presentation for each additional week (OR 1.08; 95% CI 1.04–1.11; p < 0.001), indicating that timely cancer treatment is essential for successful treatment. Patients with a substantial comorbidity burden (CCI ≥3) had an increased probability of developing late-stage disease (OR 1.37; 95% CI 1.02–1.83; p = 0.036). The research results identified socioeconomic status, geographic location, and clinical characteristics as the primary factors contributing to delays in cancer diagnosis (**Table 9**).

**Table 9:** Multivariable logistic regression.

Predictor	Adjusted OR (95% CI)	p-value
Low vs High SES	2.45 (1.70–3.52)	<0.001
Mid vs High SES	1.45 (1.02–2.05)	0.039
Rural Residence	1.60 (1.15–2.24)	0.005
Delay/Week	1.08 (1.04–1.11)	<0.001
CCI ≥3	1.37 (1.02–1.83)	0.036

### 3.10 Multivariable Cox proportional hazards model for overall survival

The table shows results from the multivariable Cox regression model, which tested various factors affecting overall survival after controlling for age, diagnosis stage, molecular subtype, and treatment adherence. The research found that people with low socioeconomic status experienced worse survival outcomes, with a hazard ratio of 1.72 (95% confidence interval, 1.32-2.24; p < 0.001). The advanced disease stage of Stage III/IV showed the highest ability to predict survival, with hazard ratios decreasing with increasing mortality risk, reaching 3.85 (95% confidence interval, 2.78-5.33; p-value < 0.001). Patients with triple-negative breast cancer showed significantly worse outcomes than patients with hormone receptor-positive subtypes because their hazard ratio reached 2.10 with a 95% confidence interval from 1.60 to 2.75 and a p-value below 0.001. The research found that treatment non-adherence showed an independent relationship with survival because the hazard ratio reached 1.65 with a 95% confidence interval from 1.24 to 2.18 and a p value below 0.001. The proportional hazards assumption was satisfied for all variables included in the Cox models. The research results demonstrate how socioeconomic disadvantage and tumor biology and adherence behaviors together create effects that impact breast cancer survival rates (**Table 10**).

**Table 10:** Multivariable Cox model for overall survival.

Predictor	Adjusted HR (95% CI)	p-value
Low vs High SES	1.72 (1.32–2.24)	<0.001
Stage III/IV	3.85 (2.78–5.33)	<0.001
Triple Negative	2.10 (1.60–2.75)	<0.001
Treatment Non-Adherence	1.65 (1.24–2.18)	<0.001

### 3.11 Multivariable Cox proportional hazards model for disease-free survival

The research found that patients with low socioeconomic status (SES) exhibited a 60% higher chance of recurrence or death when compared to patients with high SES (HR 1.60; 95% CI 1.25 2.05; p < 0.001), which reveals how socioeconomic status affects disease outcomes. The research found that an advanced stage at diagnosis (Stage III/IV) was the most dangerous factor, predicting adverse outcomes (HR 2.95; 95% CI 2.10, 4.15; p < 0.001) because it was associated with an almost threefold higher risk of disease recurrence. The research found that triple-negative tumors created worse DFS outcomes for patients (HR 1.92; 95% CI 1.48 2.50; p < 0.001) when compared to patients with hormone receptor-positive disease. The research found that patients who finished their complete treatment program achieved better DFS results (HR 0.59; 95% CI 0.45 0.78; p < 0.001) because their recurrence risk decreased by 41%. The proportional hazards assumption was satisfied for all variables included in the Cox models. The research shows how socioeconomic factors, tumor biology, and treatment adherence work together to determine long-term disease control (**Table 11**).

**Table 11:** Multivariable Cox model for DFS.

Predictor	Adjusted HR (95% CI)	p-value
Low vs High SES	1.60 (1.25–2.05)	<0.001
Stage III/IV	2.95 (2.10–4.15)	<0.001
Triple Negative	1.92 (1.48–2.50)	<0.001
Therapy Completion	0.59 (0.45–0.78)	<0.001

### 3.12 Summary of socioeconomic status–related disparities in clinical outcomes

Low SES patients showed 2.45 times greater risk for late-stage diagnosis and 1.72 times higher death risk when compared to high SES patients, while middle SES patients demonstrated moderate risk increases (OR 1.45; HR 1.28). Disease-free survival followed a similar pattern (HR 1.60 for low SES), underscoring persistent inequities in long-term outcomes. Low SES patients showed a 32% higher rate of triple-negative tumor incidence, which indicates they experience more dangerous disease patterns. Disadvantaged groups showed much lower treatment compliance rates, with low SES patients stopping therapy at 23% lower rate, which resulted in extended treatment delays of 3.6 weeks. Five-year overall survival rates differed by 18.5% between low-SES and high-SES patients, with a 24-month gap in median survival time. The research highlights socioeconomic factors as essential drivers of disparities in cancer treatment outcomes and calls for specific solutions to address them (**Table 12**).

**Table 12:** Adjusted summary of SES disparities.

Domain	Low vs High	Mid vs High
Adjusted Odds Late Stage	2.45	1.45
Adjusted HR (OS)	1.72	1.28
Adjusted HR (DFS)	1.60	1.22
Triple Negative Odds	1.32	1.12
Treatment Completion	↓23%	↓7%
5-yr OS Absolute Diff	–18.5%	–6.2%
Median OS Difference	–24 months	–10 months
Treatment Delay	+3.6 weeks	+1.4 weeks

## 4 Discussion

This paper provides a detailed analysis of the role of socioeconomic status across the breast cancer care continuum. The results indicate that socioeconomic disadvantage is associated with multiple interrelated factors, including disparities in access to healthcare, delayed diagnosis, and differences in treatment adherence and outcomes. These results emphasize the importance of structural and systemic obstacles, such as a limited healthcare access, reduced health literacy, and finances, which can have a collective impact on cancer detection and management. Previous studies have also demonstrated that socioeconomic and geographic inequalities contribute to differences in cancer incidence and stage at diagnosis (14, 15).

One of the key findings of this study is the strong association between SES and stage at diagnosis. Patients with lower socioeconomic status were more likely to present at an advanced stage of the disease, consistent with our findings, which indicated almost twice the rate of late-stage presentation in the low-SES group. This association can be attributed to a low level of taking screening programs and late-onset seeking healthcare. The trends reported are consistent with previous work, in which populations with lower SES have consistently had more advanced disease and worse outcomes (16, 17). Moreover, these differences can be enhanced by institutional and healthcare system inequalities. The fact that facilities that cater to low SES populations could struggle to uphold optimal standards of cancer care could also serve as a factor in the delayed diagnosis and ineffective results (18).

The current research demonstrated that patients with low SES showed a tendency toward more aggressive tumor biology including higher tumor grade, lymph node involvement, and elevated Ki-67 levels in socioeconomically disadvantaged populations. These results indicate clinical signs of tumor aggressiveness as opposed to causal biological impacts. This trend can be partially attributed to late detection and possible interplay of environmental exposures and socioeconomic stressors. Although psychosocial factors could contribute to the health-seeking behavior and development of the disease, it should be viewed with reservations within the framework of tumor biology (19, 20). Moreover, the socioeconomic factors at the neighborhood level can also impact the quality of care provided, which is also a contributing factor to the difference in tumor severity and care (21).

We also found substantial variation in molecular subtype distributions across SES groups. The proportion of triple-negative breast cancer was significantly increased in the low SES patients in our cohort, implying that a larger proportion of patients have more aggressive tumor phenotypes. These inequalities could be due to a complex interplay among biological susceptibility, environmental exposures, and access to healthcare. Past researches have also indicated that treatment access and outcomes also vary depending on socioeconomic status, especially in HR +/HER2- and aggressive subtypes of breast cancer (22–24).

The treatment utilization had definite socioeconomic gradients. Patients with low SES had reduced chances of accessing multimodal therapies as per the guidelines, which underscores inequalities in access to best cancer care. The results are in line with previous research showing that the disadvantaged groups tend to get inferior treatment and worse survival rates (7, 25). The socioeconomic aspects like income, education, and health care access are still the major determinants of treatment patterns and outcomes (13).

Substantial differences in SES were also found to be associated with treatment adherence in our study. These findings demonstrate that socioeconomic barriers are critical to the determination of treatment continuity and adherence, which are crucial determinants of long-term outcomes. These disparities may be caused by such factors as financial toxicity, lack of healthcare access, and inadequate support systems of patients. This implies that access and continuity of care are not only influenced by socioeconomic barriers. Investigations carried out on a population scale have identified that socioeconomic disparities play a major role in the need to adhere to breast cancer clinical guidelines and eventually affect survival rates (26). The differences in guideline-concordant therapy have been reported in aggressive forms of breast cancer, where socioeconomic status and insurance coverage determine the probability of receiving full recommended therapy (27). It can thus be necessary to address these barriers with supportive care programs and better access to healthcare in order to improve the survival differences in breast cancer.

Socioeconomic disparities strongly affect breast cancer diagnosis and treatment pathways. Patients with low SES face delays from symptom onset to diagnosis and treatment, reflecting structural and financial barriers to timely care. Similar disparities have been reported across settings, where disadvantaged groups experience delayed surgery and treatment, contributing to higher mortality (28). In low- and middle-income countries, limited access to screening and healthcare constraints further delay diagnosis and treatment initiation (29). Delayed access to care and inadequate insurance are also associated with late-stage presentation, underscoring the need to improve early access to healthcare (30).

In the present study, significant disparities in long-term survival were observed between SES groups. These differences in survival are probably the cumulative outcomes of late diagnosis and aggressive tumor features, as well as low treatment compliance. Previous research has similarly shown that neighborhood socioeconomic conditions and healthcare accessibility strongly influence treatment outcomes and survival among breast cancer patients (31). Environmental and social determinants such as healthcare resources, transportation, and social support have also been identified as important contributors to survival disparities among breast cancer survivors (32).

The multivariate analysis is another way to confirm that socioeconomic disadvantage is a contributing factor to late-stage diagnosis, even after clinical and demographic variables have been taken into account. Additional predictors included rural residence, prolonged diagnostic delays, and higher comorbidity burden. Similar findings have been reported in low-resource settings where limited awareness, low screening participation, and delayed healthcare-seeking behavior contribute to late-stage diagnosis (33). Population-based cohort studies also indicate that healthcare accessibility, socioeconomic status, and structural barriers play major roles in stage disparities in breast cancer diagnosis (34). Research conducted in sub-Saharan Africa further highlights geographic and socioeconomic inequalities as key contributors to the delay in advanced-stage detection (35).

The results of the Cox regression emphasize the combined effect of both biological (tumor stage, subtype) and socioeconomic factors on survival. The strongest predictor of mortality was advanced stage, which agrees with the existing evidence (36). Access to treatment, such as endocrine therapy and CDK4/6, has advanced treatment in hormone receptor-positive disease with beneficial outcomes (37), although triple-negative breast cancer is still linked to worse prognosis (38).

The disease-free survival analysis also indicated that the recurrence risk is influenced by a combination of socioeconomic, clinical, and treatment-related factors. Treatment compliance was identified as a critical modifiable variable, and therapy completion significantly reduced recurrence. Previous research also finds the stage and subtype of tumors to be significant predictors of outcome (39) and disease-free survival to be a surrogate for overall survival in hormone receptor-positive breast cancer (40). Altogether, the recurrence risk depends on the specifics of the tumor, the adherence, and patient-related factors (41).

Overall, the findings of this study highlight the substantial impact of socioeconomic disparities across the entire breast cancer care continuum. Patients from lower socioeconomic backgrounds were more likely to present with advanced disease, experience treatment delays, demonstrate lower treatment adherence, and ultimately exhibit poorer survival outcomes. These results emphasize the complex interaction between healthcare accessibility, tumor characteristics, and social determinants of health. Previous studies have similarly identified socioeconomic inequality as a major contributor to disparities in breast cancer incidence, treatment access, and mortality (42, 43).

Clinically and in terms of public health, the findings underscore the importance of focusing on interventions to mitigate socioeconomic disparities. Enhanced access to screening programs, increased insurance coverage, deployment of patient navigation programs, strengthened adherence support mechanisms, and improved early detection and treatment completion are strategies that can be implemented to improve early detection and treatment completion. To have fair cancer care outcomes, these structural barriers must be addressed.

### 4.1 Strengths of the study

The paper is an in-depth assessment of socioeconomic disparities in breast cancer, integrating clinical, pathological, treatment, and survival outcomes within a single analytical framework. The number of patients is quite big 800, and the distribution is even among socioeconomic groups, which supports the statistical validity of the results. The various dimensions of cancer care, including stage at diagnosis, tumor biology, treatment use, adherence patterns, diagnostic delays, and long-term survival, enable a holistic view of the impact of socioeconomic status on the overall process of care. Moreover, multivariable logistic regression and Cox proportional hazards modeling allowed the presence of major aspects of confounding factors, e.g. comorbidity burden and disease stage, thus strengthening the reliability of associations. A 72-month minimum follow-up also gave interesting long-term survival data, which reinforces the validity of the outcome assessment.

### 4.2 Limitations

Although it has strengths, the study has a number of limitations that need to be recognized. First, the retrospective design can introduce bias into the selection process and limit the ability to establish causal links between socioeconomic status and clinical outcomes. Second, the research was carried out in one oncology center (tertiary level), which could restrict the applicability of the results to other systems of healthcare or geographic areas. Third, proxies in which the socioeconomic status was categorized included income level, education and insurance coverage, which might not be wholly able to reflect the multidimensional nature of socioeconomic disadvantage. Moreover, other factors that might be considered relevant, such as lifestyle determinants, screening attendance, transport considerations, and psychosocial determinants, were not available in the medical records and thus could not be included in the analysis. Lastly, the measure of treatment adherence was based on information recorded in the clinical record, which may have underestimated instances of patient non-compliance.

### 4.3 Future recommendations

Future studies are required to be prospective and multicentric studies involving a wide population to further comprehend the socioeconomic disparity in breast cancer outcomes when used in various healthcare settings. Adding more specific socioeconomic predictors, such as occupation, household income and social support systems, could give a more precise measure of socioeconomic deprivation. Also, research on factors within the healthcare system, including the availability of screening programs, financial toxicity, access to transportation, patient navigation services, and others, might help identify modifiable barriers to care. In particular, interventional research examining targeted interventions would be useful for closing socioeconomic disparities, including community-based screening programs, patient education, financial aid systems, and adherence support measures. The outcomes for socioeconomically disadvantaged groups might also be greatly improved by integrating public health policies that increase insurance coverage and accessibility to early detection services.

## 5 Conclusion

To summarize, this paper has shown that socioeconomic status is a strong predictor of breast cancer outcomes at various levels of the disease pathway. Lower socioeconomic status patients had late diagnosis, advanced stage disease, aggressive tumor features, poorer adherence to treatment and worse survival outcomes. Socioeconomic disadvantage was an independent predictor of late-stage presentation and limited survival, even after accounting for clinical and biological factors. The findings underscore the urgency of developing targeted healthcare policies and interventions to enhance early diagnosis, reduce the time lag between treatment initiation and improvement, and improve treatment adherence among socioeconomically disadvantaged groups. These disparities must be addressed to accomplish equitable cancer care and overall increase breast cancer survival outcomes.

## Data Availability

The raw data supporting the conclusions of this article will be made available by the authors, without undue reservation.
